# Social interaction of people living with dementia in residential long-term care: an ecological momentary assessment study

**DOI:** 10.1186/s12913-024-12056-y

**Published:** 2024-12-23

**Authors:** Doris Gebhard, Leonie Lang, Marco J. Maier, Martin N. Dichter

**Affiliations:** 1 Department of Health and Sport Sciences, Technical University of Munich, Munich, Germany; 2 Protestant Education Centre Munich E.V., Munich, Germany; 3 Independent Researcher, Vienna, Austria; 4 Institute of Nursing Science, University of Cologne, Cologne, Germany

**Keywords:** Dementia, Long-term care, Social interaction, Ecological momentary assessment

## Abstract

**Background:**

The importance of social health is increasingly recognized in dementia research. For most people living with dementia, their social environment changes as the disease progresses, especially when they move into a long-term care facility. However, maintaining social interactions in the new living environment contributes significantly to health and quality of life. Staff and other residents are the most readily available interaction partners to provide this. The aim of this study is to investigate the frequency, contexts, partners and influencing factors (personal and contextual) of social interactions of people living with dementia in residential long-term care.

**Methods:**

Participants were observed for two days in 20-min slots (from 7 am to 7 pm) in 12 long-term care facilities in Germany. The Maastricht Electronic Daily Live Observation Tool (MEDLO-tool) was used for ecological momentary assessment. Age, gender, functional status, cognitive status and length of stay at the facility were recorded. Generalized linear mixed-effect models were used for the data analysis.

**Results:**

In all, 106 people living with dementia (average age: 85.16 ± 7.42 years, 82.9% female) were observed at 6134 time points. No social interaction take place in 71.9% of the observations. The place where the participants spend their time influences the occurrence of social interaction (*p* < 0.001), with a significantly higher probability of social interaction in communal spaces. Most frequently, interaction takes place with staff (43.4%), closely followed by other residents (40.9%), with the context (location, *p* < 0.001; time of day, *p* < 0.001) and functional status (care level, *p* < 0.001) influencing which of the two groups people living with dementia interact with.

**Conclusion:**

A better understanding of the context of social interactions and its influencing factors provide a basis for more targeted interventions. As the increasing staff shortage will further limit the opportunities for social interaction with staff, future concepts should focus on other residents. Meaningful activities that enable people living with dementia to co-operate and share responsibility can provide a stimulating framework for this. In addition, social assistance robots and the application of peer-mentoring/leading represent promising approaches for creating a socially interactive environment.

**Supplementary Information:**

The online version contains supplementary material available at 10.1186/s12913-024-12056-y.

## Background

The importance of social health is increasingly recognized in dementia research. The shift from a predominantly biomedical understanding of dementia to a more integrative bio-psychosocial approach is becoming internationally noticeable [1, 2]. The prioritization of social health on the international dementia research agenda [2, 3] and a growing number of studies on social factors influencing the onset and trajectories of dementia [4, 5] reflect the considerable efforts made in recent years.

In addition to empirical research, social health has also been conceptually linked to dementia. Two dementia-specific social health concepts have been presented in recent years [6, 7]. Both refer to the three dimensions of social health [8]: (a) having the capacity to fulfil one's potential and obligations, (b) the ability to manage life with some degree of independence, despite medical condition and (c) the participation in social activities. The social functioning in these three dimensions is influenced by personal and disease-related factors of a person living with dementia and is highly dependent on the structure and function of their (social) environment [6, 7]. Relevant structural factors in the social environment of people living with dementia are, for example, a person's social network [9] and opportunities for social participation [10]. Social support and social capital are examples of functional factors [11]. In addition to the structure and function of the social environment, which are objective indicators, the person’s appraisal of the quality of social contacts and the fulfilment of subjective social needs is discussed as the third environmental domain of the dementia-specific concept of social health [7]. Social connectedness and loneliness mark the two poles on the continuum of a person's subjective experience and appraisal of their social environment [12, 13]. However, social interactions are the seed of any domain of social health, the central component of social participation [10] and the basis to develop and maintain meaningful, close and constructive social relationships with others und thus experience social connectedness [12].

For most people living with dementia, their social environment changes as the disease progresses, especially when they move to a long-term care facility. Due to a relocation, contact with family, friends or neighbors may decrease, but at the same time the shared living situation with other residents, the permanently present staff and common daily routine offer new opportunities for social interaction [14–17]. This makes staff and other residents the most available interaction partners for people living with dementia in residential long-term care [18]. For this reason, the present study not only focuses on the occurrence of social interaction, but in particular on social interaction with persons within the long-term care facility, i.e. with staff and other residents.

However, studies indicate that people living with dementia experience significant barriers to social interactions and have a higher risk for social isolation [19, 20]. They face difficulties in initiating and maintaining social interactions especially when hearing loss makes communication even more challenging [19]. In addition, cognitively healthy care home residents tend to avoid social interaction with cognitively impaired residents due to cognitive and behavioral symptoms of dementia [21, 22]. Nevertheless, studies have also identified positive interactions between cognitively healthy and cognitively impaired care home residents. These interactions often occur when the cognitively healthy residents support those with dementia by enquiring about their health, advocating for them, or assisting with everyday tasks [23, 24].

Not only the other residents are important partners for social interaction, but also the staff [19]. Residents report substantial similarities in the ways they experience, define, and develop relationships with peers and staff [23, 25]. Both relationships are developed on the basis of the reaction to social interactions [25] and foster a sense of belonging and of being recognized as significant within the care facility [23]. Differences between relationships with peers and staff can be identified by examining the context of their social interaction. Residents report facility-based recreational activities, communal meals and passing time as associated with social interaction with peers [21, 25]. “Getting needs met” is reported as the prevailing context for social interaction with staff [25].

Through a salutogenic lens, it seems all the more important to focus on social interactions that take place despite the challenges outlined above, as studies indicate their health benefits. A higher frequency of social interaction was found in people living with dementia in residential long-term care to be associated with better mood [26], positive affect [27], less behavioral and psychological symptoms of dementia [28], and higher quality of life [29].

There is a growing body of research investigating social interactions of people living with dementia in residential long-term care (e.g. [26, 29–33]). Most studies focus only on selected aspects of social interactions in the everyday life, e.g. care staff-initiated interaction [30], social interaction between mutually unknown residents [31], social interaction in communal spaces [32, 33], or during certain times/activities of day [30, 32, 34]. Some of them specifically focus on quality of staff-resident care interactions [e.g. [33–35]. The few existing ecological momentary assessment (EMA) studies [18, 26, 29] provide a more naturalistic picture of the social interactions in the everyday lives, as they observe social interactions throughout the day, during all activities and in all living spaces of the care facility. These studies revealed, that people living with dementia in residential long-term care spend between 62% [29] and 83% [18] of their daytime without any social interaction and that more than twice as many social interactions take place with staff (59%) than with other residents (25%) [18]. However, none of these EMA studies investigated when, where and in what context social interactions with different partners take place.

The outlined insight into the state of research reveals that there have been considerable efforts in recent years to better understand social interactions in people living with dementia in long-term care facilities, but also that some pieces are still missing to complete the picture [20, 23]. The present study aims to address the following three currently existing research gaps: (1) Factors that influence the occurrence of social interactions, based on naturalistic everyday life and not just on selected aspects of it, (2) distribution of social interactions with other residents and staff in the different living spaces of the facility, in the course of the day, and during the most common activities, and (3) factors influencing whether people living with dementia are more likely to interact with other residents or with staff. This study contributes to research on social interactions of people living with dementia in residential long-term care by answering the following research questions within two subjects of interest:

1. Occurrence of social interactions:
  a How frequent are social interactions in everyday life and what is the proportion of different interaction partners?
  b Do the (i) personal characteristics of age, gender, global functional status and cognitive status and the (ii) contextual factors of the care home's location, location within the facility, and the daytime influence the occurrence of social interactions?
2. Social interactions with other residents and staff:
  a How are social interactions with other residents and staff distributed in terms of location within the facility, daytime and type of activity?
  b Do the (i) personal characteristics of age, gender, global functional status and cognitive status and the (ii) contextual factors of the care home's location, location within the facility, and the daytime influence whether interaction with other residents or with staff is more likely?

The following hypotheses were formulated to address research questions 1b and 2b:

- H1: The (i) personal characteristics of age, gender, global functional status and cognitive status and the (ii) contextual factors of the care home's location, location within the facility, and the daytime are significant predictors of the occurrence of social interactions.
- H2: The (i) personal characteristics of age, gender, global functional status and cognitive status and the (ii) contextual factors of the care home's location, location within the facility, and the daytime are significant predictors of whether social interactions take place with other residents or with staff.

## Methods

### Study design

The current observational study uses observer-rated ecological momentary assessment to record the social interactions in the everyday lives of people living with dementia in residential long-term care. The use of ecological momentary assessment builds an extensive, real-time picture of social interactions as it documents the direct context in which social interaction naturally occurs during the day [36, 37]. For cognitively impaired people in particular, this has advantages over survey methods that are based on retrospective thinking about their social contacts [38]. An observational application of ecological momentary assessment can be a suitable option for cognitively impaired people, as it allows the daily routine to be documented without the person having to constantly recall and complete the documentation.

This study emerged from the collaboration of two larger studies on the psychosocial health and quality of life of people living with dementia in residential long-term care in Germany: CaResource (Shaping long-term care, promoting health, empowering people) and Qol-DEM II (Evaluation of measurement methods for the assessment of quality of life and daily life of people with dementia).

### Setting and sample

The data was collected in a total of 12 care homes in two German federal states, five in Bavaria (region of Munich, relatively high socioeconomic status) and seven in North Rhine-Westphalia (region of Oberhausen, relatively low socioeconomic status) [39]. Although long-term care insurance in Germany is regulated nationwide, there are individual deviations depending on the federal state. Two facilities are located in rural areas, ten in urban areas. Between 62 and 208 residents live in the facilities, with the average number of residents being 113.9 ± 44.7. The selected facilities represent a convenient sample and are therefore not representative. The average care facility in Germany has between 80 and 100 places, although there are also significantly smaller facilities (< 10) and larger ones (> 300) [40]. All facilities can be characterized as traditional care homes. In 11 of the participating care homes, one ward each took part in the study; in one care home, two different wards took part. In each ward, a convenience sample of 7 to 10 participants were recruited by asking the care manager for residents fulfilling the following inclusion criteria: (a) dementia diagnosis noted in the care documentation, (b) not cared for in bed, as this limits the opportunities for social interactions [41], (c) living in the facility for at least two weeks. All participants, as well as their guardians (in case of legal representation) provided written informed consent. Situational dissent, i.e. a verbal or non-verbal indication of unwillingness to participate in study procedures, was respected at any time during the observations. All data was collected between October 2020 and June 2022. The period of data collection was extended due to COVID-19 access restrictions in residential long-term care facilities.

## Measures

### Participant characteristics

The global functional status was determined by the care level of the German five-level system [42] (higher levels indicate a higher impairment of independence) which was taken from the participants' care documentation. Cognitive functioning was assessed with the Dementia Screening Scale (DSS), which measures seven items on the domains of memory and orientation from the perspective of nursing staff in long-term care facilities [43]. The DSS total score varies between 0 and 14 (a higher score indicates more severe cognitive impairment). The internal consistency of the DSS proved to be good with Cronbach’s alpha = 0.94 and it is easier to apply and less time consuming compared to performance-based instruments. The DSS demonstrated high correlations with other established screening instruments like Mini-Mental Status Examination. In a validation study in German nursing homes, the DSS showed better criterion validity than global diagnosis-related staff assessments [43].

Age, gender and the duration of stay within the facility was taken from the participants' care documentation.

### Social interactions and their contexts

The German version of the Maastricht Electronic Daily Live Observation tool (MEDLO-tool) was used to investigate social interactions and its contexts. The original Dutch MEDLO-tool was developed in 2016 [44] and translated into German in the preparation phase of the Qol-DEM II project in 2018 [45]. The tool allows the observation of daily life activities (32 categories), engagement in activities (five categories), physical effort (7 point Likert scale), location (five categories), level of social interaction (five categories), type of social interaction (five categories), social interaction with whom (five categories), interaction with the physical environment (dichotomous categories), mood (7 point Likert scale) and agitation (5 point Likert scale). The original MEDLO-tool demonstrated face validity, acceptable inter-rater reliability scores between 0.5 and 1 (Kappa or weighted Kappa scores) and feasibility in research practice [44].

The MEDLO-tool was applied in its entirety and the following variables were used for this study:

- Occurrence of social interaction: Any type of verbal or non-verbal communication between the participant and one or more persons is scored as social interaction, regardless of whether the contact is reciprocated or not.
- Interaction partner: staff, other residents, family and/or friends, others
- Location of interaction: communal spaces (communal area on the ward, communal area off the ward, outside), private spaces (own room, bathroom/toilet)
- Activity in which the social interaction takes place: different activities that reflect the everyday life of people living with dementia in long-term care facilities, such as eating and drinking, caring activities, watching television, reading, and having a conversation. In addition to the specific activities, the two activities of sitting/lying down (the resident is sitting or lying down but is awake, there is no activity taking place) and conscious resting (e.g. the resident is deliberately placed in bed by the care staff) can be scored.

Since this study focuses only on the occurrence of social interaction and not on the type of interaction, the results for the variable “type of social interaction” are only outlined in the Additional file 1 (Supplementary Table 1).

### Procedure

Data were collected on two consecutive days by previously trained research staff with several years of experience in the field of long-term care. All participants were observed from 7 a.m. to 7 p.m.. The observations took place in 20-min slots, within each participant was observed for one minute, after which the observer scored the variables. The researchers observed the participants where they were at the time and documented the situation without influencing it (e.g. through interaction). The order in which the participants were observed was randomly generated by the MEDLO software for each observation time point. The observation day was organized in three blocks (morning 07:00–11:30, afternoon 11:30–16:00, and evening 16:00–19:00), with breaks in between.

### Analysis

Generalized linear mixed-effects models were used to account for the repeated measurements (where multiple measurements are nested within participants at level 2). A “forward stepwise” procedure was applied, i.e. model selection was done by iteratively testing and adding statistically significant terms to the model until no significant terms remained. To identify factors that influence social interaction, the occurrence of social interaction (yes vs. no) was used in the first model as the dependent variable in a logistic regression with a random effect. In the second model, the same was done to identify factors that influence social interaction partners, using the partner in social interaction (other residents vs. staff) as the dependent variable. The following independent variables were considered in the two models (along with all interactions): (a) location of the care home (Bavaria vs. North Rhine-Westphalia), (b) participant characteristics (gender, age centered at 85, care level, DSS score centered at 7), (c) location of observation (communal spaces vs. private spaces), and (d) daytime of observation (morning, afternoon, evening).

Descriptive analysis were made using SPSS Statistics (IBM Corporation, Version 29.0. Armonk, NY), generalized linear mixed-effects models were performed in R [46] with the package lme4 [47].

## Results

### Sample characteristics

In all, 106 participants were observed resulting in 6134 momentary assessments. Table 1 presents the sample characteristics.

**Table 1:** Sample characteristics

Variable	Participants (*n* = 106)
Age, years, mean ± SD	85.16 ± 7.42
Gender, female (%)	82.9
Duration of stay, months, mean ± SD	29.46 ± 26.47
Functional status, care level, mean ± SD	3.48 ± 0.9
Cognitive status, DSS score, mean ± SD	6.62 ± 3.36

*SD* Standard Deviation, *DSS* Dementia Screening Scale

### Frequency and partners of social interaction

No social interaction takes place during more than two thirds of the observations (71.9%). The values for the individual facilities range between 60.2 and 81.3%. The observed social interaction most frequently occurs with staff (43.4%), closely followed by interaction with other residents (40.9%). Social interaction with family or friends rarely takes place in the participants’ everyday lives (3.9%); 11.7% of social interaction occurs with other interaction partners or a combination of different interaction partners.

### Context of social interaction with other residents and staff

#### Locations of social interaction

**Fig. 1:**
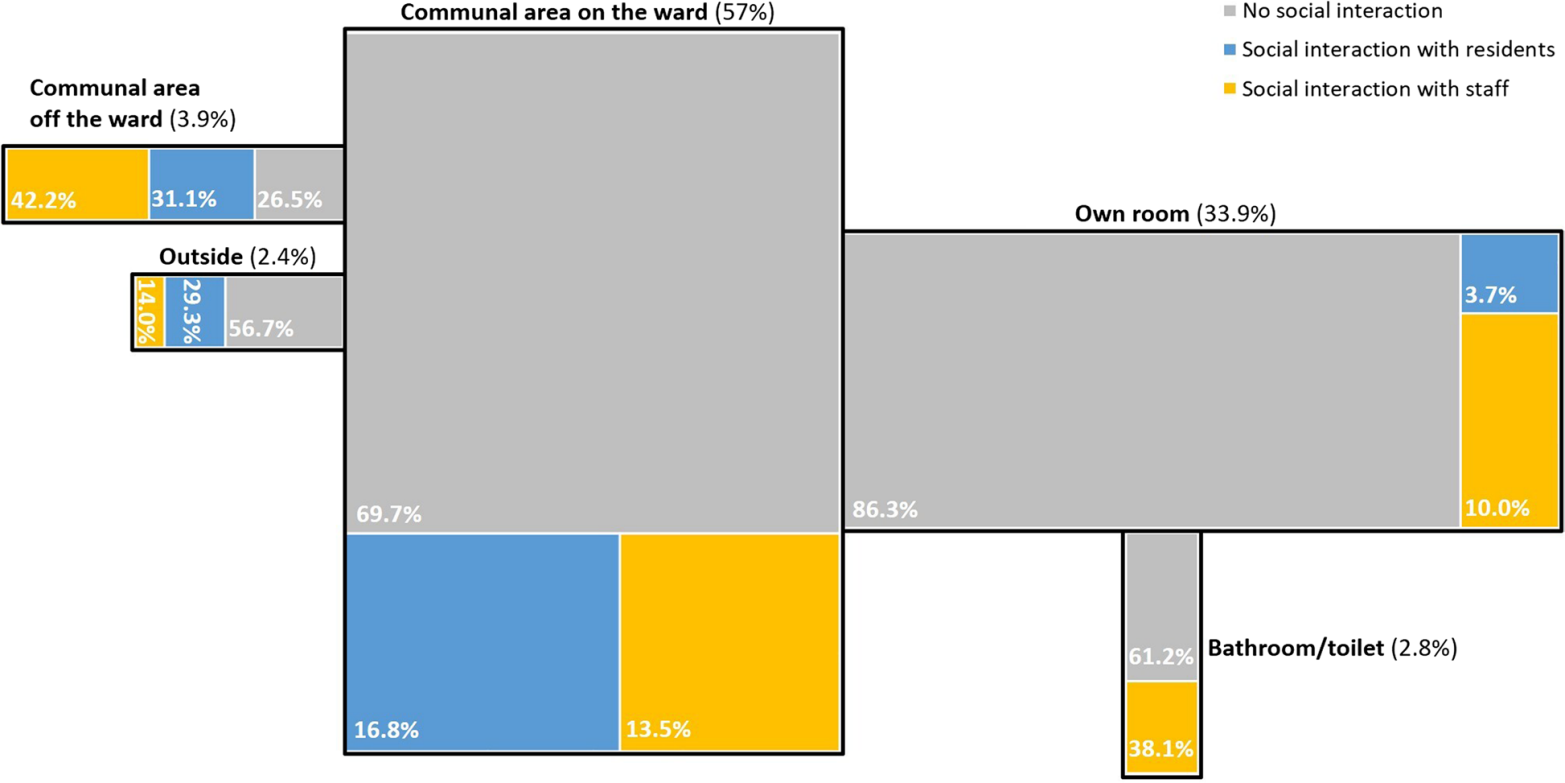
Locations of social interaction with other residents and staff, *n* = 5153. Note: The figure shows a tree-map diagram using a schematic floor plan of a residential long-term care facility

Figure 1 illustrates a schematic floor plan of a residential long-term care facility (communal spaces: communal area on the ward, communal area off the ward, outside; private spaces: own room, bathroom/toilet). The size of the areas shown in Fig. 1 corresponds to the proportion of observations made there (as also indicated by the percentages). For each of the areas, the distribution of observations in which there was no social interaction, interaction with other residents and interaction with staff is shown in different colors.

The participants spend most of their time in the communal area on the ward (57.0%), followed by their own room (33.9%). In both locations, there is no social interaction during most of the observation moments. However, social interaction can be observed more frequently in communal spaces (33.4%) than in the residents' private spaces (16.0%). Interactions with other residents occur more frequently in the communal area on the ward and outside. The staff is the predominant interaction partner in the residents’ own room, the residents’ bathroom/toilet and the communal area off the ward.

#### Daytime of social interaction

**Fig. 2:**
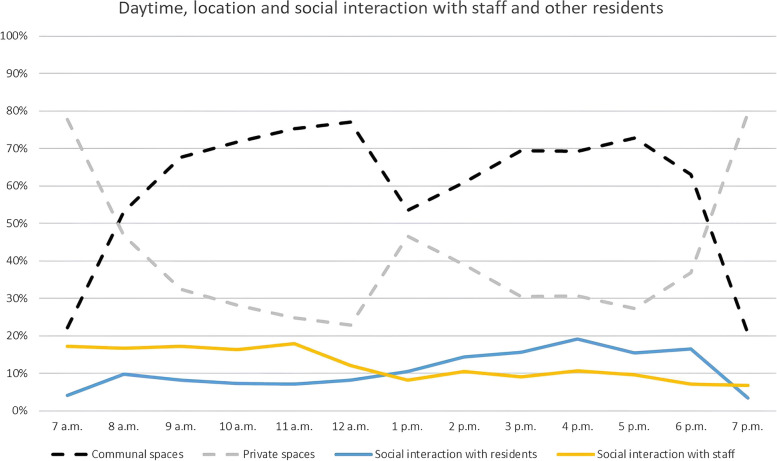
Daytime, location and social interaction with staff and other residents, *n* = 1221

The percentage of observed social interaction during the day is between 20% (7 p.m.) and 35.7% (4 p.m.). Figure 2 shows the proportion of social interactions that took place with other residents and staff during the course of the day in combination with the distribution of locations (communal spaces vs. private spaces). In the early morning, residents remain in their private living environment and social interaction mainly takes place with the staff. In the midmorning, participants spend their time more frequently in communal spaces, but the staff remains the primary interaction partner until noon. In the early afternoon, the amount of time spent in private spaces increases slightly. In the afternoon and early evening, participants mainly spend their time in communal spaces, and at this time of day, they mainly interact with other residents. Between 6 and 7 p.m., the frequency of observations in private spaces increases again and the proportion of interactions with other residents decreases in parallel.

#### Activities and social interaction

Table 2 shows the proportion of social interaction during the ten most frequently observed activities and how the social interaction with staff and other residents is distributed.

**Table 2:** Top 10 activities and social interaction with other residents and staff, *n* = 5153

Top 10 activities ( % of observations)	Proportion of social interaction during the activity	Social interaction	
		Staff	Residents
Sitting/lying down (33.1%)	12.6%	39.6%	60.4%
Eating and drinking (15.8%)	22.1%	57.8%	42.2%
Resting (11.1%)	1.8%	33.3%	66.7%
Walking (8.5%)	35.2%	61.8%	38.2%
Having a chat (5.0%)	100%	14.0%	86.0%
(Self) care activity (4.6%)	51.8%	100%	0%
Watching television, listening to the radio (4.6%)	4.3%	30.0%	70%
Reading, writing, crossword puzzle (1.9%)	7.1%	14.3%	85.7%
Playing cards, playing a game, doing a puzzle (1.7%)	95.2%	68.0%	32.0%
Music/singing (1.4%)	74.7%	83.3%	16.7%

In almost half of the observations, no specific activity was detected, as the participants spent their time sitting/lying down (33.1%, the person is awake but not active) or resting (11.1%, conscious rest, e.g. the person is deliberately put to bed by staff), which is accompanied by low rates of social interaction (12.6% and 1.8% respectively). Compared to the total percentage of social interaction during the day (28.1%), a higher degree of social interaction takes place during five activities. Interactions with staff are observed more frequently than with other residents when eating and drinking, going for walks and during structured social activities. Every social interaction that takes place during (self) care activities occurs with staff. Other residents were observed as the predominant interaction partner during periods of inactivity, while watching television or listening to the radio, and reading or solving crosswords. Conversations (as the main activity) are observed much more frequently with other residents than with staff.

#### Factors influencing social interaction

The final logistic regression model includes only location as a factor influencing the occurrence of social interactions (see Table 3), with social interactions more likely to occur in communal spaces. All variables that were not included in the model are presented in the Additional file 2 (Supplementary Table 2).

**Table 3:** Factors influencing social interaction

	Estimate	Std. Error	z value	Pr( >|z|)
(Intercept)	-0.778	0.088	-8.865	< .000 001***
Location (private spaces)	-1.069	0.092	-11.599	< .000 001***

Standard deviation of random effect = 0.778; ***p < 0.001

#### Factors influencing the partners in social interaction

Table 4 shows the final logistic regression model. The daytime, the location and the care level of people living with dementia proved to be influencing factors on interaction partners. In the afternoon and evening social interaction is much more likely with other residents than with staff, in private spaces interaction with staff is more likely than with residents, and at care level 5, the likelihood of social interaction with other residents is significantly lower than social interaction with staff.

**Table 4:** Factors influencing the partners in social interaction

	Estimate	Std. Error	z value	Pr( >|z|)
(Intercept)	-0.682	0.390	-1.751	0.0799
Daytime (afternoon)	1.087	0.166	6.532	< .000 001***
Daytime (evening)	1.534	0.211	7.260	< .000 001***
Location (private spaces)	-1.740	0.246	-7.073	< .000 001***
Care level 3	0.370	0.432	0.857	.391 590
Care level 4	-0.469	0.434	-1.079	.280 460
Care level 5	-1.775	0.584	-3.039	.002 380**

Standard deviation of random effect = 1.124; ** *p* < 0.01, *** *p* < 0.001

## Discussion

This observational study uses 6134 momentary assessments to investigate the frequency, contexts, partners and influencing factors of social interaction in the everyday lives of people living with dementia in residential long-term care. The results show that the participants spend most of their time without social interaction, as this only takes place for around a quarter of the day. The place where people living with dementia spend their time influences the occurrence of social interaction, with a significantly higher probability of social interaction in communal spaces. Most frequently, interaction takes place with staff, closely followed by other residents, with the context (location, time of day) and functional status (care level) influencing which of the two groups people living with dementia interact with.

### Frequency, context and influencing factors of social interaction

The frequency of social interaction in this study (28.1%) is slightly lower than in the studies by Beerens and de Boer et al. [29] (32%) and Beerens and Zwakhalen et al. [26] (33.5%), but much higher than observed by Adlbrecht et al. [18] (17.1%). However, rates of social interaction observed in this study are far below those reported for non-residential long-term care. People living with dementia interact socially in 68.8% of observations in regular day care centers and in 81.2% in farm-based day care [48]. Firstly, this reflects the stronger focus of day care facilities on psychosocial aspects of care by providing opportunities for social interaction through a variety of activities [48, 49]. Secondly, the social benefit of farm-based care, which has already been demonstrated for people living with dementia in residential long-term care, is becoming evident [50]. The underlying effect that leads to increased social interaction in green care farms is the provision of meaningful activities which encourage community building through co-operation and shared responsibility (e.g. household and farm chores) [51]. Looking at the most frequently observed activities in the current study and the proportion of social interaction during these, it becomes clear that this type of activity rarely occurs in everyday care home life, which could be one reason for the low level of social interaction. Thus, the finding that meaningful daytime activities and company are the two most frequently unmet needs in care homes [52] can be quantitatively supported by the present study.

The relevance of communal spaces as the most important place for social contact [21] was confirmed by the current findings. Over the course of the day, the times around shared meals seem to increase the presence in the communal spaces (peaks at noon and 5 p.m.) and thus social interaction. This reflects the important role of shared meals in everyday care home life, which not only influence the daily routine but also the opportunities for social interaction [22, 53]. Moreover, the results indicate that the place where people living with dementia spend their time during the day is a relevant factor that influences the occurrence of social interaction. The design of communal spaces could play an important role in determining how much time people living with dementia spend there and whether the environment is more conducive or obstructive to social interaction. This assumption is supported by the results of Adlbrecht et al. [54], who identified the built environment of long-term care facilities as a factor influencing social interaction. Smaller and more homely environments were found to be beneficial for promoting social behavior in people living with dementia [55]. However, the results clearly show that most of the time spent in communal spaces is not used for social interaction, which is consistent with the findings of Morgan-Brown et al. [32]. Other studies also suggest that it is quite common for people living with dementia to sit quietly in communal spaces, “minding their own business” [53, 56]. Measures to promote social interaction can therefore not simply aim to increase the time spent in communal spaces, but must also enable people living with dementia to take advantage of the opportunities for social interaction that these spaces offer. In addition to the built environment, studies have also identified other characteristics of the facility, such as the care philosophy and the organizational environment [54], as well as personal factors, such as functional status and age, as influencing factors for social interaction [19, 38]. Based on previous research with other populations [57, 58] it can be presumed that personality traits also influence the social behavior of people living with dementia. However, this has hardly been researched to date [59] and could be a subject for future studies.

### Frequency, context and influencing factors of partners in social interaction

The ranking by frequency of the observed interaction partners is consistent with the results from previous studies [18], with the most consistent finding being a low level of social interaction with family and friends. Even though the proportion of social interaction with family and friends in the present study appears to be very low at 3.9%, it is still higher than in the study by Adlbrecht et al. [18] (2.5%) focusing on Austrian nursing homes. The focus of the present study, however, is on social interaction with persons within the long-term care facility, i.e. staff and other residents.

### Social interaction with staff

Compared to Adlbrecht et al. [18], the current study found a considerably lower percentage of interactions with staff (43.4% vs. 58.7%). It is possible that the timing of the data collection influenced the frequency of interaction with staff, as the observations took place during and after the COVID-19 pandemic, which was associated with increased workloads for care home staff [60]. Rationing care is widely discussed as a direct response to the excessive workload of nursing staff [61], not only in the context of the COVID-19 pandemic, but certainly exacerbated by it. A qualitative study [62] reveals that due to limited resources, care home staff is forced to use the available time as efficiently as possible. Task-orientated direct care, such as feeding, washing, toileting and administering medication, is given the highest priority, while time for conversation and meeting the psychosocial needs of residents has the lowest priority [62]. Dalby Kristiansen et al. [63] show that staff use also task-oriented direct care for social interaction with people living with dementia, which is supported by our results, since social interaction with staff takes place in more than half of the (self) care activities. Furthermore, factors associated with a higher probability of interaction with staff indicate a connection with care activities, since participants with the highest care needs are more likely to have social interactions with staff.

However, the existing staff shortages in long-term care, the high work demands and the predicted deterioration of the situation [64] will further limit opportunities for social interaction with staff in the future [19]. This makes it even more important to make better use of the readily available social resource of other residents.

### Social interaction with other residents

This study found a higher rate of social interaction with other residents than the study of Adlbrecht et al. [18] (40% vs. 25.1%). In contrast to social interaction with staff, social interaction with other residents is more likely to occur in communal spaces than in private spaces. One reason for this could be the limited quality of the relationships between people living with dementia and their peer residents. Studies on the quality of social relationships have shown that residents tend to perceive each other as friendly acquaintances and that close relationships are rare [21, 22]. Thus, private spaces may be too intimate to invite other residents and are rather perceived as a safe retreat [65]. Most social interactions with other residents appear to arise from shared daily routines and are incidental, which is largely consistent with the results of previous studies [21, 25, 53].

The proportion of social interaction with other residents increases throughout the day and peaks between 4 and 6 p.m., presumably during dinner. The abrupt retreat to private rooms immediately after dinner may be due to the discomfort some people living with dementia experience when sitting with other residents they do not know very well [65] or it may be facilitated by what people believe is expected of them as care home residents [21]. Psychosocial group activities were only documented in 3% of the observations and are not characterized by social interaction with peer residents but with the staff. The available results from the literature are inconsistent regarding the question of whether structured activities provide good opportunities for social interaction between residents [25]. This may indicate that the respective roles of staff and residents in implementing psychosocial activities are crucial for the occurrence of social interaction. Staff can either take on a facilitating role to enable residents to interact with each other [38], or they can assign the residents a passive and consuming role that inhibits peer interaction [66]. The crucial role of staff in facilitating social contact is also anchored in the German expert standard for relationship management in the care of people living with dementia [67]. The identification of people living with dementia who need support in establishing and maintaining relationships, and the implementation of targeted activities to promote relationships, is defined in this guideline as a central task of care staff [67].

### Implications

Social interactions with other residents, staff and family or friends differ in their qualities, characteristics and the psychosocial outcomes they support [23]. The two intervention approaches outlined deliberately focus on fostering interaction with other residents, as they represent the most accessible social resource with untapped potential for greater utilization in the future.

Socially assistive robots were found to be feasible and effective to improve social interaction in people living with dementia in long-term care facilities [68, 69]. The so-called companion robots either have the shape of pets (such as a cat, dog or seal) or are humanoid in design and can interact through sounds and movements [68]. Within a group of residents, these robots represent a common reference object that can be interacted with directly, but which can also be talked about. Moreover, companion robots of the next generation have advanced artificial intelligence, which makes it possible to adapt the interaction to the needs of the interaction partners [70]. This type of robot is still in the development phase, but it shows new possibilities for addressing the needs and requirements of social interaction of people living with dementia in a more personalized way.

A second approach that focuses more on the social empowerment of people living with dementia is peer mentoring. Theurer et al. [71, 72] trained people living with dementia in residential long-term care and volunteers as mentors to improve the social engagement of vulnerable groups, such as new residents or residents who refuse to participate in the social activities offered. In this program, people living with dementia are empowered to take on the role of a mentor by visiting the mentees in pairs. Skrajner et al. [73] have demonstrated that it is also possible to empower people living with dementia to lead group activities for their peers in long-term care facilities. The results of this pioneering work on peer mentoring approaches are promising in terms of their impact on psychosocial health and their feasibility, but further research is needed on both outcomes [71–73].

The present study focuses on the occurrence of social interaction, which is an objective marker of the structure of the social environment of people living with dementia [7]. However, it is important to consider that a high number of social interactions does not necessarily protect against loneliness, as loneliness reflects a subjective assessment of the social environment in terms of the quality of social contacts [74]. Future research should merge the investigation of objective and subjective markers of social health in mixed-method designs to provide a more holistic picture of the social world of people living with dementia in residential long-term care.

### Strengths and limitations

The greatest strength of the current study is the use of an ecological momentary assessment to obtain a real-time picture of social interaction in the daily lives of people living with dementia that is not biased by the inaccuracy of retrospective or proxy data collection methods. Furthermore, this is the first study to analyze the factors influencing social interaction based on naturalistic everyday life (not just on selected aspects of it) and to distinguish in the analyses between interaction with other residents and with staff. In addition, all observations were carried out by qualified members of the research team with experience in long-term care. A detailed user manual of the MEDLO-tool was available in case of uncertainties.

However, the present study also has some relevant limitations. The observations took place during and after the COVID-19 pandemic, although always at times when the restrictions on social contacts were very relaxed or not applied. Nevertheless, the COVID-19 pandemic may have affected the results. It can be assumed that the social distancing rules not only had an immediate effect on the social behavior of people living with dementia in long-term care, but also longer-term effects, such as a reduction in trust in social relationships or a change in the sense of belonging to the group of residents. These changes could have led to a decrease in social interactions during the data collection period. Although the number of momentary assessments is rather large, only two observation days per participant have been included. Several days with a deliberate distribution over working days and weekend days would certainly paint a richer picture and allow more conclusions to be drawn about everyday life as a whole. The long-term care facilities and participants were selected by convenient sampling, which limits the generalizability of the results. The generalizability of the results is further limited by the exclusion of people who are cared for in bed. It can be assumed that the inclusion of this group of people would have influenced the study results, particularly with regard to the frequency of interaction with other residents, as they spent most of their time in their private rooms. Future studies should particularly examine the social interaction of people who are cared for in bed as they are at a heightened risk of social isolation due to their limited mobility. Understanding their specific needs and barriers to participation is crucial for developing targeted strategies that promote their social inclusion. Furthermore, we did not differentiate by care concepts or size of long-term care facility, which could also be an influencing factor for the occurrence of social interactions. Nesting measurements not only within participants, but also within the facilities would better address the supposed influence of the facility characteristics in the analysis.

## Conclusion

Residential long-term care provides a unique environment of social interaction for people living with dementia. The current study draws a real-time picture of how, when and with whom social interactions naturally take place during the day and identifies influencing factors. People living with dementia in residential long-term care spend most of the day being socially inactive. The occurrence of social interactions is significantly influenced by where participants spend their time, with communal spaces proving to be favorable. Although interactions with staff and other residents occur almost equally frequently, the contextual factors differ. Interaction with staff takes more likely place during the morning, in private spaces and with people living with dementia who have high care needs. Interactions with other residents occur more likely later in the day, in communal spaces of the care home and as part of shared daily routines. As the increasing staff shortage will further limit the opportunities for social interaction with staff, future concepts for promoting social interaction should focus on peers. Meaningful activities that promote community building through co-operation and shared responsibility can create a framework to increase the opportunity for social interaction between people living with dementia and their peer residents. Concepts on how this can be realized outside of farm-based care should be developed in future research. Social assistance robots are a promising approach for creating a socially interactive environment for people living with dementia in residential long-term care. Future research should focus on where and how artificial intelligent robots can be used to improve human interaction between residents in particular. Peer mentoring and peer leading are resource-orientated and empowering approaches to encourage people living with dementia to interact socially. Initial pioneering work in this area shows the potential of the approaches but more research is now required to put these preliminary results on a sound evidence base.

However, determining the best approaches to promote social interaction requires a more personalized perspective, given the diverse needs of individuals living with dementia in residential long-term care. Further research is essential to better understand the unique social interaction needs and preferences within this population. Developing social interaction profiles could provide a foundation for tailored interventions, both with and without the integration of artificial intelligence. Additionally, mixed-methods studies that combine objective and subjective measures of social health could offer deeper insights into the social experiences of people living with dementia, enhancing our ability to support their well-being in residential care settings.

## Supplementary Information

Additional file 1. Type of social interaction, doc, presenting a supplementary table showing the percentages of different types of social interaction in general and in relation to interactions with other residents and with staff.

Additional file 2. Factors influencing social interaction, not significant variables, .doc, presenting a supplementary table on the variables that were not significant in the forward stepwise procedure of model selection.

## Data Availability

Datasets generated and analyzed during the current study are available from the first author upon reasonable request.

## Abbreviations

DSS: Dementia Screening Scale
MEDLO-tool: Maastricht Electronic Daily Live Observation tool
SD: Standard Deviation
