# Supplementary material for: Social interaction of people living with dementia in residential long-term care: an ecological momentary assessment study
Source: BMC Health Serv Res. 2024 Dec 23;24:1640. doi: 10.1186/s12913-024-12056-y (PMC11668022; doi:10.1186/s12913-024-12056-y)
Supplement: Supplementary file 1 — Additional file 1. Type of social interaction, doc, presenting a supplementary table showing the percentages of different types of social interaction in general and in relation to interactions with other residents and with staff. [file 12913_2024_12056_MOESM1_ESM.docx]

| Type of social  interaction | All social interactions | Social interaction  with staff | Social interactions with other residents |
| --- | --- | --- | --- |
| Negative | 1.8% | 1.8% | 2.0% |
| Neutral | 60.0% | 51.0% | 75.1% |
| Positive | 38.2% | 47.8% | 22.9% |

**Additional file 1: Type of social interaction**

Supplementary Table 1: Type of social interaction

Note: Negative = the summarized categories of negative restrictive and negative protective; Positive = the summarized categories of positive care and positive social
