# Supplementary material for: Social interaction of people living with dementia in residential long-term care: an ecological momentary assessment study
Source: BMC Health Serv Res. 2024 Dec 23;24:1640. doi: 10.1186/s12913-024-12056-y (PMC11668022; doi:10.1186/s12913-024-12056-y)
Supplement: Supplementary file 2 — Additional file 2. Factors influencing social interaction, not significant variables, .doc, presenting a supplementary table on the variables that were not significant in the forward stepwise procedure of model selection. [file 12913_2024_12056_MOESM2_ESM.docx]

**Additional file 2: Factors influencing social interaction, not significant variables**

Supplementary Table 2: Factors influencing social interaction, not significant variables

|  | Df | AIC | LRT | Pr(>Chi) |
| --- | --- | --- | --- | --- |
| None |  | 5501.4 |  |  |
| Location (care home) | 1 | 5501.9 | 1.4733 | 0.22483 |
| Gender | 1 | 5499.8 | 3.5787 | 0.05853 |
| Age | 1 | 5503.4 | 0.0322 | 0.85763 |
| DSS score | 1 | 5500.4 | 3.0030 | 0.08311 |
| Daytime | 2 | 5501.6 | 3.7539 | 0.15306 |
| Care level | 3 | 5501.5 | 5.9243 | 0.11535 |

Note. Df = Degrees of freedom, AIC = Akaike information criterion, LRT = Likelihood-Ratio-Test
